# Massive Pleural Fluid Collection in Adult Nigerians

**DOI:** 10.1155/2016/6946459

**Published:** 2016-06-29

**Authors:** Kelechi E. Okonta, Emmanuel O. Ocheli, Peter D. Okoh

**Affiliations:** Cardiothoracic Surgery Unit, Department of Surgery, University of Port Harcourt Teaching Hospital, PMB 6173, Port Harcourt, Rivers State, Nigeria

## Abstract

*Background*. There are no available literatures on massive pleural effusions (MPE) in our country.* Aim*. To determine the aetiology of MPE and compare the mortality rate between malignant and nonmalignant MPE in adult Nigerians.* Methods*. A prospective study of all the patients diagnosed with nontraumatic pleural fluid collections for one year in two tertiary federal hospitals in Southern Nigeria. A total of 101 consecutive patients with pleural fluid collections were studied. Diagnoses were made by clinical features and laboratory and radiological investigations.* Results*. Forty-eight patients (47.5%) had MPE with a mean age of 43 years ± 14.04 and 35 were females. Thirty patients (62.5%) were diagnosed with nonmalignant conditions (21 from pulmonary tuberculosis (PTB) and 9 from other causes). Haemorrhagic pleural collections were from malignancy in 12 (30.8%) and from PTB in 6 (15.4%). Straw-coloured collections were from malignancy in 9 (23.1%), from PTB in 8 (20.1%), and from posttraumatic exudative effusion in 3 (7.7%). Compared with nonmalignant MPE, patients with malignant collections had higher mortality within 6 months (8/18 versus 0/30 with a* P* value of 0.000).* Conclusion*. The presentation of patients with nontraumatic haemorrhagic or straw-coloured MPE narrows the diagnosis to PTB and malignancy with MPE cases being a marker for short survival rate.

## 1. Introduction

Massive pleural fluid collection is commonly caused by malignancy [1–3] and has an incidence of 11.2–12% of all the pleural effusions [2, 3] and thus remains a definite health care problem. In our region, the aetiology and occurrence of massive pleural fluid collection have remained largely undetermined and therefore not known. However, a study done over a decade ago, considering all the pleural fluid collections, showed that infective aetiologies such as pulmonary tuberculosis and empyema thoracis were responsible for the pleural fluid collections in over 90% and malignant aetiology contributed to less than 10% of the collections [4]. Massive pleural fluid collection by definition is the effusion which on chest radiograph appears as complete or near-complete opacification of the ipsilateral thorax; that is, the opacification is over two-thirds of the hemithorax [1, 5]. On the bases of aetiology, massive pleural fluid collections can be classified into malignant massive pleural fluid collection and nonmalignant massive pleural fluid collection [2, 3] with malignant massive pleural fluid collection portending a poorer prognosis [3]. As patients with malignant massive pleural fluid collection have shorter survival, the aim of the treatment, therefore, is to improve the quality of life by ensuring the amelioration of symptoms such as dyspnoea, cough, and chest pain [6] and prevention of recurrence while treatment for those with infective causes is aimed at cure and also at preventing recurrence.

## 2. Methods

Massive pleural fluid collection was diagnosed by the use of plain chest roentgenograms which when it showed opacity of about two-thirds and above made the diagnoses. Furthermore, the pleural aspirates and pleural biopsies were subjected to cytological and histological analyses for suspected cases of malignant effusions and the pleural aspirates tested for acid fast bacilli.

This is a prospective study of all the patients who had symptomatic pleural fluid collection diagnosed between June 2012 and July 2014 by making use of clinical features, radiological parameters, and pleural aspirate for cytology and acid fast bacilli testing. Informed consent was obtained at the time of institution of chest tube. The term massive pleural fluid collection is the effusion which on posteroanterior chest roentgenogram shows complete or near-complete opacification of the ipsilateral thorax; that is, the opacification is at least two-thirds of the hemithorax [1, 5]. All the patients who were 18 years of age and above and had chest tube instituted in the thoracic units were included while excluding patients with acute traumatic pleural fluid collections (haemothorax), patients who refused chest tube insertion, and patients who died before the insertion of chest tubes.

Two tertiary health centers in the southern part of the country were used. A total of 101 consecutive patients were diagnosed with pleural fluid collection that required chest intubation. All closed tube thoracostomies were done under local anaesthesia (1% xylocaine) using appropriate intercostal spaces. Patients with malignant massive pleural fluid collection, in addition, had chemical pleurodesis done before extubation. The patients were discharged home or to their primary units when the postextubation chest roentgenograms showed adequate drainage of fluid and significant lung reexpansion. The age, sex, clinical features, laboratory investigation results, radiological findings, diagnosis, and the treatment outcome of the pleural effusion were entered into a pro forma.

Patients were seen in 2-week time in the clinic and if no major complaints are seen in the next 1 month, then they were seen twice monthly, three times monthly, and 6 times monthly with chest roentgenogram at each visit to assess recurrence and mortality.

The data were analyzed using computer based statistical software Minitab version 16. Excel was used for descriptive statistics (the mean and standard deviation) and significance was defined as *P* < 0.005 by Chi-square test.

## 3. Results

Between June 2012 and May 2013, 101 consecutive patients were treated for pleural fluid collections out of whom 48 patients (47.5%) had massive pleural fluid collections with a mean age of 43 years + 14.04; 35 were females and 13 were males with a ratio of 2.7 : 1. All the patients presented with the following cardinal symptoms: dyspnoea in 97.7%, cough in 79.1%, chest pain in 48.8%, and weight loss in 39.5%. Eighteen patients (37.5%) had malignancy as follows: 11 had metastatic breast cancer, 3 had primary lung cancer, 3 had metastatic ovarian cancer, and 1 had metastatic prostate cancer. Thirty patients (62.5%) were diagnosed with nonmalignant conditions as follows: pulmonary tuberculosis in 21 (44.9%), posttraumatic effusion in 3, parapneumonic effusion in 3, amoebic liver abscess in 2, and biventricular failure in 1 (Table 1). The breakdown, based on the colour of the pleural fluid collections, was mainly haemorrhagic or straw-coloured in 39 patients: haemorrhagic pleural collections were from malignancy in 12 (30.8%) and from pulmonary tuberculosis in 6 (15.4%) while straw-coloured effusions were from malignancy in 9 (23.1%), pulmonary tuberculosis in 8 (20.1%), posttraumatic exudative pleural effusion in 3 (7.7%), and biventricular heart failure in 1 (2.6%) (Table 2). The information for 48 patients regarding mortality and recurrence was got at clinic during follow-up visits or through phone calls. One patient with massive pleural fluid collection from breast cancer had recurrent effusion which necessitated another cycle of treatment. Eight out of 18 patients who were diagnosed with malignant massive pleural fluid collection died within 6 months of follow-up. The mortality in malignant massive pleural fluid collection when compared with mortality in nonmalignant massive pleural collection for the 30 patients followed up shows that mortality in malignant massive pleural fluid collection is higher (8/18 versus 0/30 with a *P* value of 0.000).

## 4. Discussion

To the best of our knowledge, this is the first study giving the aetiology and mortality rate of massive pleural fluid collection in the West African setting and certainly in the country. 47.5% of the recorded cases of massive pleural fluid collection, from this review, are adjudged high when compared with the results of other studies [2, 3]. The high occurrence is an indication that most of the patients present late for health care and also indicates the problem of delay in making diagnosis in patients with pleural effusion that could lead to massive pleural fluid collection. The pathological process of massive pleural fluid collection involves the progressive accumulation of fluid in pleural space [5] which if not promptly treated can be massive.

Massive pleural fluid collection can be caused by a wide range of clinical conditions [4, 5, 7–9] that can be grouped into malignant and nonmalignant causes [1, 2]. Studies from Spain showed that malignancy was responsible for over half of the causes of massive pleural fluid collection seen in that area [2, 3, 10]. In that study, lung cancer tops the list of aetiology closely followed by breast cancer, while infective causes like pulmonary tuberculosis and parapneumonic effusion accounted for less than one-third of nonmalignant massive pleural fluid collections [2, 3]. From this review, there is a reversal in aetiologies, as nonmalignant causes accounted for about two-thirds of the causes of massive pleural fluid collection (with infectious causes like pulmonary tuberculosis and parapneumonic effusion constituting half) while malignancy formed slightly above one-third of all the aetiology. The causes of massive malignant pleural effusions were mainly from breast cancer followed by primary lung cancer and primary ovarian cancer. In areas with high incidence of tuberculosis, the common cause of pleural effusion is pulmonary tuberculosis followed by malignancy [4, 11, 12] which, by extension, is the common cause of massive pleural fluid collection. Massive pleural collection occurred most in adults, because malignancy, especially breast cancer, is common in this age group [13].

Equally, massive pleural fluid collection may be an unusual presentation in some clinical condition. For instance, the first presentation of systemic lupus erythematosus in a patient could be massive pleural fluid collection and this could form an important clinical point of diagnosis even when there are no overt stigmata [9]. It may be an unusual presentation in patients with prostate cancer [8]. In trauma, the multiple rib fracture can lead to the formation of exudative pleural effusion especially in the elderly [7] instead of haemothorax by some other mechanism. One of the patients from the review had massive pleural fluid collection from metastatic prostate cancer while 3 patients with trauma also had exudative massive pleural fluid collection.

In terms of symptomatic presentation, dyspnoea is the most common and important diagnostic symptom in patients with massive pleural fluid collection. The pathogeneses of this symptom are compressive atelectasis of the lung, diaphragmatic depression, and reduced chest compliance [5, 14]. Almost all the patients in our review had dyspnoea as presenting complaints. This is because when there is large or massive effusion which has been chronic, the patient commonly experiences shortness of breath [6].

Massive pleural fluid collection narrowed the diagnosis to a smaller number of aetiologies [2]. Importantly, the combination of nontraumatic haemorrhagic and straw-coloured massive pleural fluid collection, in our setting, was able to narrow our diagnosis to pulmonary tuberculosis and malignancy as aetiologies. The practical utility of colour of pleural collection in making diagnosis has not been previously documented in the literatures for these clinical conditions.

Malignant massive pleural fluid collections are associated with worse survival than nonmalignant massive pleural fluid collection and massive malignant pleural effusion is thus a poor prognostic marker for malignancy [3]. The finding that about half of the patients who were diagnosed with malignant massive pleural fluid collection died between 3 and 6 months after chest tube drainage and chemical pleurodesis lends support to this assertion. It is probable that if these patients are followed up up to 12 months, higher mortality would be recorded in malignant massive pleural fluid collections.

## 5. Conclusion

The presentation of an adult patient with nontraumatic haemorrhagic or straw-coloured massive pleural fluid collection in this subregion narrows the diagnosis to pulmonary tuberculosis and malignancy with malignant massive pleural fluid collection indicating shorter survival. It is instructive to state that pulmonary tuberculosis and breast cancer in women account for the great burden of MPE seen in our setting and will be necessary to intermittently screen these patients with a chest roentgenogram.

## Figures and Tables

**Table 1:** The aetiology of massive pleural fluid collections.

Aetiology	Total
Pulmonary tuberculosis	21 (44.9%)
Metastatic malignancy^*∗*^	18 (37.5%)
Parapneumonic effusion	3 (6.3%)
Posttraumatic exudative effusion	3 (6.3%)
Amoebic liver abscess	2 (4.2%)
Biventricular ventricular failure	1 (2.1%)

^*∗*^Metastatic breast cancer: 11, primary lung cancer: 3, metastatic ovarian cancer: 3, and metastatic prostate cancer: 1.

**Table 2:** The 39 patients with haemorrhagic or straw-coloured pleural collections.

Causes	Haemorrhagic	Straw-coloured
Pulmonary tuberculosis	6 (15.4%)	8 (20.1%)
Metastatic malignancy	12 (30.8%)	9 (23.1%)
Other	0 (0%)	4 (10.3%)
